# Direct Expression of Fluorinated Proteins in Human Cells for ^19^F In-Cell NMR Spectroscopy

**DOI:** 10.1021/jacs.2c12086

**Published:** 2023-01-05

**Authors:** Lan B. T. Pham, Azzurra Costantino, Letizia Barbieri, Vito Calderone, Enrico Luchinat, Lucia Banci

**Affiliations:** † CERM—Magnetic Resonance Center, Università degli Studi di Firenze, Via Luigi Sacconi 6, 50019Sesto Fiorentino, Italy; ‡ Consorzio Interuniversitario Risonanze Magnetiche di Metallo Proteine—CIRMMP, Via Luigi Sacconi 6, 50019Sesto Fiorentino, Italy; § Dipartimento di Chimica, Università degli Studi di Firenze, Via della Lastruccia 3, 50019Sesto Fiorentino, Italy; ∥ Dipartimento di Scienze e Tecnologie Agro-Alimentari, Alma Mater Studiorum—Università di Bologna, Piazza Goidanich 60, 47521Cesena, Italy

## Abstract

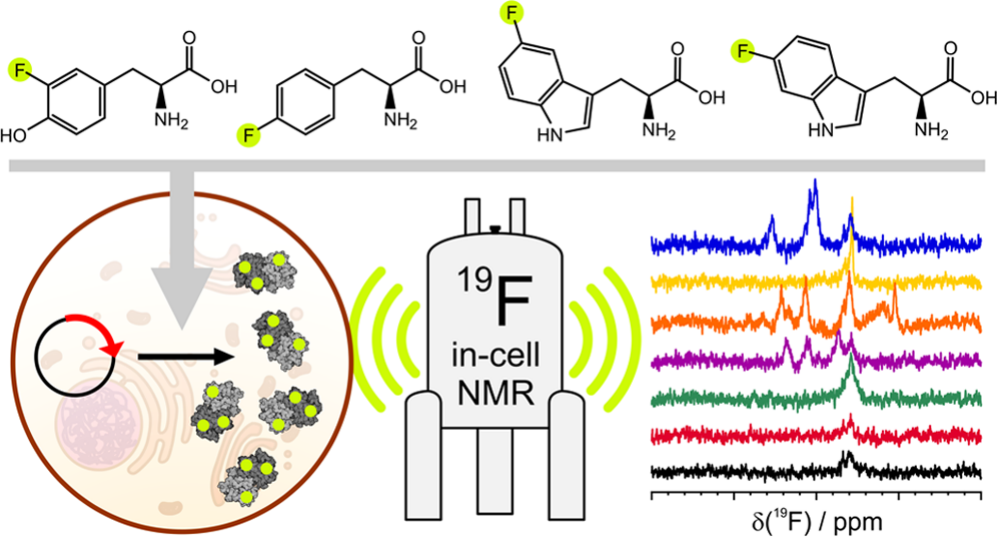

In-cell NMR spectroscopy is a powerful approach to study protein structure and function in the native cellular environment. It provides precious insights into the folding, maturation, interactions, and ligand binding of important pharmacological targets directly in human cells. However, its widespread application is hampered by the fact that soluble globular proteins often interact with large cellular components, causing severe line broadening in conventional heteronuclear NMR experiments. ^19^F NMR can overcome this issue, as fluorine atoms incorporated in proteins can be detected by simple background-free 1D NMR spectra. Here, we show that fluorinated amino acids can be easily incorporated in proteins expressed in human cells by employing a medium switch strategy. This straightforward approach allows the incorporation of different fluorinated amino acids in the protein of interest, reaching fluorination efficiencies up to 60%, as confirmed by mass spectrometry and X-ray crystallography. The versatility of the approach is shown by performing ^19^F in-cell NMR on several proteins, including those that would otherwise be invisible by ^1^H-^15^N in-cell NMR. We apply the approach to observe the interaction between an intracellular target, carbonic anhydrase 2, and its inhibitors, and to investigate how the formation of a complex between superoxide dismutase 1 and its chaperone CCS modulates the interaction of the chaperone subunit with the cellular environment.

## Introduction

Structural studies of biological macromolecules are typically carried out in vitro on purified samples, in which the molecules are separated from their physiological environment. To fully understand their biological function, the in vitro data must be integrated and validated with structural and functional information directly obtained in the native environment. In this respect, in-cell NMR spectroscopy stands out as the most suitable technique to study macromolecules at atomic resolution in living cells.^1,2^ Indeed, high-resolution multidimensional NMR studies in various bacterial and eukaryotic cells, both in solution and in frozen samples, have provided unique insights into how the cellular environment modulates the structure and function of proteins as well as nucleic acids through molecular crowding, diffuse interactions, chemical modifications, and homeostasis of ions and cofactors.^3−8^ In addition, in-cell NMR allows screening drug candidates for binding to an intracellular target,^9,10^ hence potentially filling an outstanding gap between in vitro lead optimization and preclinical studies in drug development, where many promising compounds fail due to the lack of target engagement in cellular or animal models.

Despite these premises, the approach still suffers from technical limitations, namely, the low sensitivity of NMR spectroscopy, short sample lifetime, and, in solution, signal loss due to line broadening effects, which challenge its application to a broader range of pharmacological targets. While sensitivity and sample lifetime can be respectively improved by employing stronger magnetic fields^11^ and flow bioreactors,^12−14^ line broadening is an intrinsic effect of the cellular environment on the target macromolecules. In cells, molecules that diffusely interact with abundant components and/or specifically bind to large-molecular size partners undergo severe line broadening due to increased transverse relaxation, regardless of their original molecular size, leading to the loss of signals in the ^1^H-detected NMR spectra, typically employed to observe isotope-labeled macromolecules in living cells.

^19^F NMR spectroscopy can overcome such limitation. ^19^F is a sensitive NMR-active nucleus with 100% natural abundance, is highly sensitive to changes in the surrounding chemical environment, and is absent in native biological molecules. As such, ^19^F incorporation in proteins or small molecules has been widely used in NMR applied to medicinal chemistry.^15^ When applied to in-cell studies, ^19^F provides NMR spectra with virtually no background signals, hence facilitating the identification of the signals of interest and the analysis of lineshape and chemical shift changes, even for slow-tumbling molecules.

^19^F in-cell NMR has been successfully employed to analyze the tumbling and dynamics of proteins in bacteria and in oocytes,^16−19^ and to observe nucleic acids in oocytes and human cells.^20−22^ Recently, the ability of ^19^F NMR to detect highly interacting proteins, which would otherwise be invisible, was demonstrated in human cells.^23^ In all of the above works, proteins labeled with fluorinated amino acids (FAAs) were recombinantly expressed in bacteria and were either analyzed directly in bacterial cells or purified for delivery into oocytes or human cells. However, the existing protocols for FAA incorporation in bacteria, with the notable exception of 5-fluoro-tryptophan synthesis from a precursor,^24^ require the use of specific auxotrophic strains or inhibitors of amino acid synthesis.^15^ Furthermore, protein delivery into human cells needs to be carefully optimized for each protein and might be incompatible with proteins that are sensitive to unwanted chemical modifications in vitro, or prone to precipitate in concentrated solutions. As a robust alternative to protein delivery methods,^5,25,26^ protein expression has been extensively employed for NMR studies in human cells.^10,27^ This methodology bypasses bacterial expression, purification, and delivery; it allows several uniform and amino acid-selective isotopic labeling schemes, albeit at the expense of some isotopic cellular background in the resulting NMR spectra, and is broadly applicable to both globular and unfolded soluble proteins.^11,28,29^

Here, we report an alternative approach for studying fluorinated proteins in human cells by NMR, which relies on the direct protein expression and incorporation of FAAs in human cells. We show that several fluorinated aromatic amino acids can be easily incorporated into the protein of interest, which is then readily detected in 1D ^19^F in-cell NMR spectra with minor interference from ^19^F cellular background. We tested this approach on a series of proteins with different molecular sizes and different folding properties. Broad signals from ^1^H NMR-invisible proteins can be detected, and structural and functional insights can be obtained when investigating intracellular protein–ligand and protein–protein interactions. The potential of this approach to study proteins having diffuse interactions with other cellular components, together with its time- and cost-effectiveness, will expand the range of applications of in-cell NMR in cellular structural biology and drug development research.

## Materials and Methods

### Gene Constructs

Vectors encoding α-synuclein (αSYN, NP_000336.1), carbonic anhydrase 2 (CA2, NP_000058.1), superoxide dismutase 1 (SOD1, NP_000445.1), DJ-1 (NP_009193.2), the second domain of the copper chaperone for SOD (CCS-D2; CCS 84-234, NP_005116.1),^3,10,11,30,31^ the N-terminal domain of heat shock protein 90 (Nt-HSP90; HSP90 9-236, NP_005339.3, unpublished work), and empty vector (pHL-empty) were obtained from the pHLsec vector^32^ after removing the secretion sequence, as described in previous works.

### Protein Expression in Human Cells

The vectors were transiently transfected and expressed in HEK293T (ATCC CRL-3216), following the protocol described by Barbieri et al.^28^ HEK293T cells were cultured in a T75 flask (Greiner Bio-One), and an in-house prepared Dulbecco’s modified Eagle’s medium (DMEM) was used for the expression of fluorinated proteins. All compositions of the in-house DMEM including nonfluorinated or fluorinated amino acids were prepared according to the compositions of commercial high-glucose DMEM. At the time of transfection, the vectors, except pHL-αSYN, were delivered into cells using polyethylenimine (PEI, Sigma-Aldrich) with ratio 1:2 (25 μg DNA: 50 μg PEI). For αSYN expression, 5 μg of pHL-αSYN was combined with 20 μg of pHL-empty in 2.5 mL of DMEM, before the addition of 2.5 mL of PEI-DMEM solution. For the co-expression of SOD1 and CCS-D2, 12.5 μg of each vector (1/2 of 25 μg DNA) were mixed and added with PEI solution as the latter. Upon the application of PEI-DNA complex, HEK293T cells were incubated at 37 °C, 5% CO_2_, and in the in-house DMEM containing nonfluorinated amino acids, 2% (v/v) of fetal bovine serum (FBS, Life Technologies) and 100 U/mL or μg/mL penicillin–streptomycin (Life Technologies), respectively. At a selected time post-transfection (switch time, ST, specified for each experiment in the Results section), the medium was replaced with fresh in-house DMEM containing one of the following fluorinated amino acids at the same concentration as the commercial medium: 6-fluoro-l-tryptophan (6FW, 80 μM), 5-fluoro-l-tryptophan (5FW, 80 μM), 4-fluoro-l-phenylalanine (4FF, 400 μM), or 3-fluoro-l-tyrosine (3FY, 600 μM). All target proteins were expressed for a total of 48 h. During the expression of SOD1, CCS-D2, and CA2, 10 μM ZnSO_4_ was supplemented to the medium.

### ^19^F In-Cell and Cell Lysate NMR Spectroscopy

After 48 h of expression, the cells were harvested, resuspended in 180 μL of NMR buffer (DMEM, 70 mM HEPES, 90 mM glucose, and 20% (v/v) D_2_O), and then transferred to a 3 mm Shigemi tube as detailed in the previous protocol.^28^ Before inserting the sample into the NMR instrument, the cell suspension was gently centrifuged to form a soft pellet in the tube. For checking the cell viability before and after NMR experiments, 10 μL of cell suspension was aliquoted out and diluted to an optimal concentration for live/dead cell assessment by trypan blue staining with an automated cell counter (LUNA-II, Logos Biosystem, Inc.).

To analyze the target proteins in cell lysates, the cells were recollected and lysed after the in-cell NMR experiments following the aforementioned protocol.^28^ Cell pellets were separated from the NMR buffer by centrifugation, and subsequently lysed in 150 μL of phosphate-buffered saline (PBS, Life Technologies) by freeze–thaw method. The soluble cell lysates were obtained from whole cell lysates by centrifugation and were analyzed by ^19^F NMR spectroscopy with 10% (v/v) of D_2_O.

All ^19^F in-cell NMR experiments were carried out at 310 K on a 14.1 T (600 MHz ^1^H) Bruker Avance III, which was equipped with a room-temperature SEL-HP probe and tuned at 564.6 MHz for ^19^F detection. A single 90° pulse immediately followed by FID acquisition was employed (zg Bruker pulse program). A set of four 28 min spectra with 1280 scans each and an interscan delay of 1 s was recorded on each sample, for a total acquisition time of 112 min. The same set of spectra was recorded on the supernatants collected after the in-cell NMR experiments to check for protein leakage, and on the corresponding cell lysates. The ^19^F NMR spectra were processed and analyzed in TopSpin 4.1.1 (Bruker). The four 1D spectra were processed with 10 Hz exponential line broadening, phase-corrected, and summed together, followed by a polynomial baseline correction applied to the sum spectrum. The last step was necessary to remove a strong baseline distortion that arose from polytetrafluoroethylene (PTFE) components inside the probe. The ^19^F chemical shift scale was referenced to trichlorofluoromethane (CFCl_3_) by setting the signal of trifluoroacetic acid (TFA) in an external reference sample to −76.55 ppm. Reference ^19^F NMR spectra of FAAs were recorded with 1024 scans on 5 mm samples containing 130 μM of each pure FAA in PBS buffer.

### Protein Quantification by SDS-PAGE

The expression levels of proteins incorporating each FAA with 24 h switch time (ST) were estimated by SDS-polyacrylamide gel electrophoresis (SDS-PAGE). The cells from T75 flasks were lysed in 150 μL of PBS. The soluble lysates were then collected and diluted 20-, 40-, and 80-fold; 6 μL of sample at each dilution, containing 100 mM Tris-HCl pH 6.8, 2% (w/v) sodium dodecyl sulfate (SDS), 12.5% (v/v) glycerol, 0.1% (w/v) bromophenol blue, and 50 mM dithiothreitol (DTT), was loaded and analyzed by SDS-PAGE using pre-cast gels (Bio-Rad), at 200 V for 30 min. In each SDS-PAGE analysis, four protein standard mixtures containing BSA and CA2 with different concentrations (0.05, 0.1, 0.15, and 0.2 mg/mL) were also included to generate linear regression standard curves. The gels were then stained with Coomassie dye (ProBlue Safe Stain, Giotto Biotech), imaged by ChemiDoc XRS (Bio-Rad), and analyzed by ImageJ.^33^ The protein expression levels were estimated by densitometry based on the standard curves.

### Incorporation Efficiency Analysis by Mass Spectrometry

The incorporation efficiency of 3FY in CA2 and αSYN, and of 6FW in CA2 and SOD1 was evaluated by mass spectrometry. HEK293T cells were cultured in T25 flasks (Greiner Bio-One). At the end of the expression, the cells were collected and lysed in 150 μL of PBS. The soluble cell lysates were subsequently separated by centrifugation and loaded on an SDS-PAGE gel as described above. The gel bands containing target proteins were excised and digested with either trypsin or chymotrypsin following a published protocol.^34^ The extracted peptides were subsequently examined by nano-liquid chromatography coupled to high-resolution tandem mass spectrometry, equipped with a nano-electrospray ionization source (nLC-nESI-HRMS/MS), using an LTQ Orbitrap hybrid mass spectrometer (Thermo Fisher Scientific). The peptide separation by nLC was operated following a linear gradient of elution solvent (80% (v/v) acetonitrile and 0.1% (v/v) formic acid, LC-MS grade, Merck). The gradient was increased accordingly to 2% of elution solvent in 2.30 min, to 30% in 176 min, to 48% in 70.15 min, and to 90% in 19.30 min. MS/MS data were acquired in data-dependent mode, where an HRMS full scan (from 280 to 2000 *m*/*z*, at 100,000 nominal resolution and 1 × 10^6^ of target value) was conducted and immediately followed by a second MS analysis of the seven most intense ion precursors (>500 a.u.) found in the survey scan. The acquired data were queried on human database (NCBI) using Mascot 2.4 (Matrix Science) to verify the identification of target peptides and proteins. The percentage of FAA incorporation in each peptide was calculated by the following equation
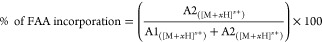
where [M + *x*H]^*x*+^ are the ionized species of the target peptide; *x* is the protonation state; A1 is the chromatographic peak area of [M + *x*H]^*x*+^ ion of the target peptide without FAA; and A2 is chromatographic peak area of [M + *x*H]^*x*+^ ion of the target peptide with FAA. For each peptide having various molecular ions with high abundance, the average of percentages of different generated species was obtained.

### 6FW-CA2 Crystallization, Data Collection, and Structure Calculation

6FW-CA2 was expressed from HEK293T as described above, with a 24 h ST, and purified by affinity chromatography by adapting a previously reported protocol.^35^ Briefly, the lysates from three identical T75 flasks were pooled, diluted to 3 mL with binding buffer (20 mM Tris, pH 8), loaded on a 1 mL Ni-NTA column equilibrated with binding buffer, and washed with 3 mL of binding buffer. Elution was carried out with 3 mL steps of increasing concentrations of imidazole in binding buffer. CA2 was eluted at high purity with 10 mM imidazole. The protein was placed in 10 mM HEPES, pH 6.8, and subsequently concentrated. Protein crystals were obtained by sitting drop vapor diffusion, by adding an aliquot of 2 μL of protein solution (15 mg/mL in 10 mM HEPES, pH 6.8) to 2 μL of reservoir buffer (2.9 M ammonium sulfate, 0.1 M Tris-HCl, pH 8.0) and stored at 20 °C. The dataset was collected in-house, using a BRUKER D8 Venture diffractometer equipped with a PHOTON III detector, at 100 K; the crystal used for data collection was cryo-cooled using 10% ethylene glycol in the mother liquor. The crystal diffracted up to 1.6 Å resolution, but the structure has been refined at 1.7 Å: it belongs to space group *P*2_1_ with one molecule in the asymmetric unit (consistent with the vast majority of CA2 entries deposited on the PDB), a solvent content of about 50%, and a mosaicity of 0.3°. The data were processed using the program XDS,^36^ reduced and scaled using XSCALE,^36^ and amplitudes were calculated using XDSCONV.^36^ The structure was solved by molecular replacement using a published crystal structure of CA2 (PDB 1CA2).^37^ The successful orientation and translation of the molecule within the crystallographic unit cell was determined with MOLREP.^38^ The refinement and water molecule fitting were carried out using PHENIX with TLS restraints.^39^ In between the refinement cycles, the model was subjected to manual rebuilding using COOT.^40^ The quality of the refined structure was assessed with MOLPROBITY.^41^ Data processing and refinement statistics are shown in Table S4. Coordinates and structure factors have been deposited in the PDB under the accession code 8B29.

## Results

### ^19^F In-Cell NMR of Human Proteins Incorporating Various Fluorinated Amino Acids

The incorporation of FAAs was achieved by replacing a chosen amino acid with the corresponding FAA in the expression medium so that HEK293T cells transiently transfected with the gene of interest could utilize it for protein synthesis. Following the observation of a drastic decrease in the expression levels of target proteins in cells supplemented with FAA-containing media immediately after transfection (Figure S1), a medium switch time (ST) of 24 h post-transfection was introduced, during which the cells were kept in normal medium to allow DNA internalization and transcription to take place without further stress. The medium was then replaced with the FAA-containing medium to allow the FAA incorporation into the expressed protein.

**Figure 1:**
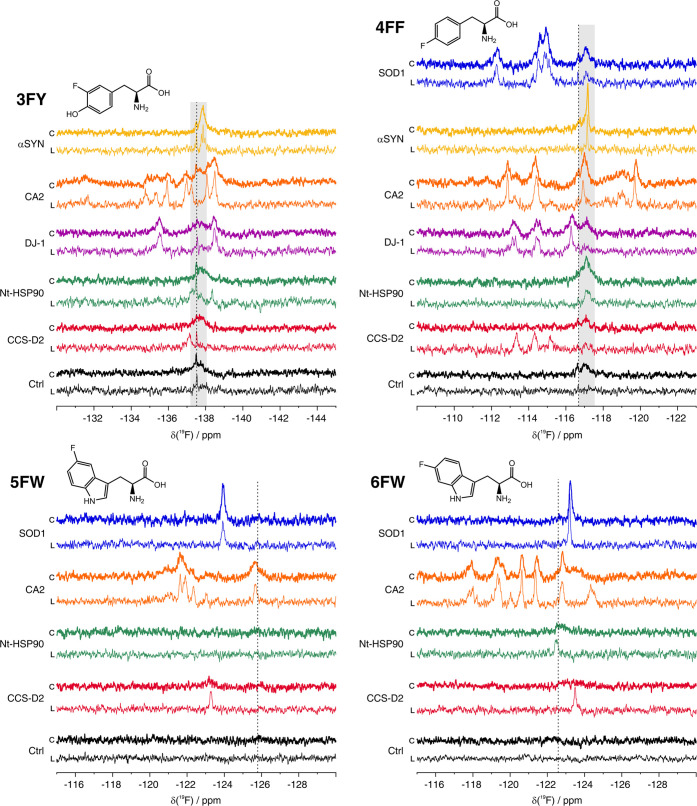
In-cell and lysate ^19^F NMR of proteins incorporating different ^19^F-amino acids. 1D ^19^F NMR spectra of HEK293T cells (c, thick line) expressing different proteins in media supplemented with 3FY (top left), 4FF (top right), 5FW (bottom left), and 6FW (bottom right) with ST = 24 h, and the corresponding lysates (l, thin line). Ctrl: control spectra acquired on HEK293T cells transfected with pHL-empty and the corresponding lysates. The regions containing the background signal of 3FY and 4FF are shown as light gray bands; the chemical shift of each free FAA is marked with a dotted line (see also Figure S2).

We tested this approach on a set of soluble human proteins: α-synuclein (αSYN), copper-zinc superoxide dismutase (SOD1), carbonic anhydrase isoform 2 (CA2), the deglycase DJ-1, the N-terminal domain of heat shock protein 90 (Nt-HSP90), and the SOD-like domain of the copper chaperone for SOD (CCS-D2). The aromatic amino acid composition for each protein is reported in Table 1. Each protein was overexpressed in the cytoplasm of HEK293T with different FAAs (3FY, 4FF, 5FW, and 6FW), resulting in protein concentrations in the corresponding cell lysates ranging from 100 to 360 μM (Figure S2 and Table S1). Due to protein overexpression and to the high sensitivity of the ^19^F nucleus, the intracellular fluorinated proteins were readily detected even using a room-temperature probe designed for ^1^H detection (see the Materials and Methods section). 1D ^19^F NMR spectra of each sample were acquired on intact cells and on the corresponding cell lysates (Figure 1). The cell viability was overall above 90 and 85% before and after NMR experiments, respectively, and the ^19^F NMR spectra of the extracellular medium showed only contributions from the free FAAs, confirming that no protein leakage had occurred (Figure S3). Control cells transfected with an empty plasmid allowed assessing the ^19^F background signals arising from the incorporation of FAAs into other cellular proteins. 3FY and 4FF incorporation resulted in signals centered at ca. −137.5 and at ca. −116.8 ppm, respectively, whereas no background signals were observed with 5FW and 6FW incorporation. Such discrepancy correlates with the much lower abundance of tryptophan residues in the proteome compared to tyrosine and phenylalanine, and with the lower concentration of tryptophan in the cell medium (see the Materials and Methods section). In the lysate spectra, the 3FY and 4FF background signals are greatly reduced (Figure 1), suggesting that their in-cell signals arise from insoluble components and/or organelles (e.g., nuclei and mitochondria), which are removed from the cleared cell lysate. The free amino acids 3FY and 4FF were also detected in the in-cell and lysate spectra at −137.5 and −116.7 ppm, respectively (Figure 1), as well as in the extracellular medium (Figure S3). The latter indicates that residual free FAAs leak from the cells during the NMR experiments; leakage of free 5FW and 6FW may have occurred below the detection limit and was not observed.

**Table 1:** Molecular Weight and Aromatic Amino Acid Composition of the Proteins Analyzed in This Study

	molecular weight (kDa)	number of phenylalanines	number of tyrosines	number of tryptophans
αSYN	14.5	2	4	0
SOD1	16	4	0	1
CA2	29.2	12	8	7
DJ-1	19.9	3	3	0
Nt-HSP90	25.6	10	7	1
CCS-D2	16	3	1	1

Overall, most of the overexpressed proteins gave rise to ^19^F signals that did not match with the cellular background (Figure 1). Both 3FY- and 4FF-αSYN resulted in a single peak, due to the low chemical shift dispersion typical of the NMR signals of unfolded proteins. The signals of αSYN were sharper and better resolved in the lysate spectra (Figure S4), consistent with previous reports.^16,42^

On the contrary, signals from globular proteins were reasonably well resolved and clearly detected in the in-cell NMR spectra, as it was observed for SOD1, CA2, and DJ-1, which are known to tumble freely in the cytoplasm (Figure 1). Notably, 5FW-CA2 gave rise to very different signal patterns with respect to 6FW-CA2 in the in-cell NMR spectra. This indicates that changing the position of the fluorine atom in the same residue can significantly report different chemical environments in the protein structure.

Strikingly, both Nt-HSP90 and CCS-D2, which experience strong interactions with other cellular components^30^ [unpublished work], making them undetectable via ^1^H- detected NMR experiments, could be detected by ^19^F in-cell NMR. 4FF-Nt-HSP90 gave rise to a prominent broad resonance (ca. −116.2 to −117.6 ppm), higher than the background signals in the control spectrum, while 6FW-Nt-HSP90 resulted in a weak broad signal rising above the baseline (ca. −122.4 to −123.2 ppm), corresponding to the only tryptophan residue of Nt-HSP90 (Figure 1). 5FW-Nt-HSP90 was not detected in either cells or lysate, despite being equally overexpressed (Figure S2 and Table S1), suggesting that 5FW might perturb the folding state of Nt-HSP90 causing the formation of soluble aggregates. In the case of CCS-D2, while 3FY- and 4FF-CCS-D2 did not give rise to in-cell resonances other than the cellular background, 5FW- and 6FW-CCS-D2 displayed very broad and low-intensity signals (ca. −122.7 to −123.6 ppm and ca. −122.3 to −124.0 ppm, respectively, see Figure 1). Despite the low intensity, these two resonances were confirmed by multiple repeated experiments and could be unambiguously assigned to the only tryptophan residue of CCS-D2, owing to the absence of cellular background signals of 5FW and 6FW. The in-cell signals of 5FW- and 6FW-CCS-D2, as well as 4FF- and 6FW-Nt-HSP90, were also confirmed in the ^19^F NMR spectra of the cell lysates (Figure 1). Therefore, ^19^F NMR allowed the observation of previously undetectable proteins. However, this outcome varied with each combination of expressed protein and FAA analog employed, likely due to the fact that the ^19^F lineshape depends on the local side-chain dynamics, which are highly protein- and position-dependent.

### Optimization of the FAA Incorporation Protocol

Based on the successful incorporation of different FAAs and their detection by in-cell ^19^F NMR, we sought to optimize the incorporation protocol by evaluating the expression levels and the incorporation efficiency of SOD1, αSYN, and CA2 labeled with 3FY and 6FW, as a function of different medium switch times (8, 16, and 24 h). The total expression time of 48 h was kept constant in all experiments.

**Figure 2:**
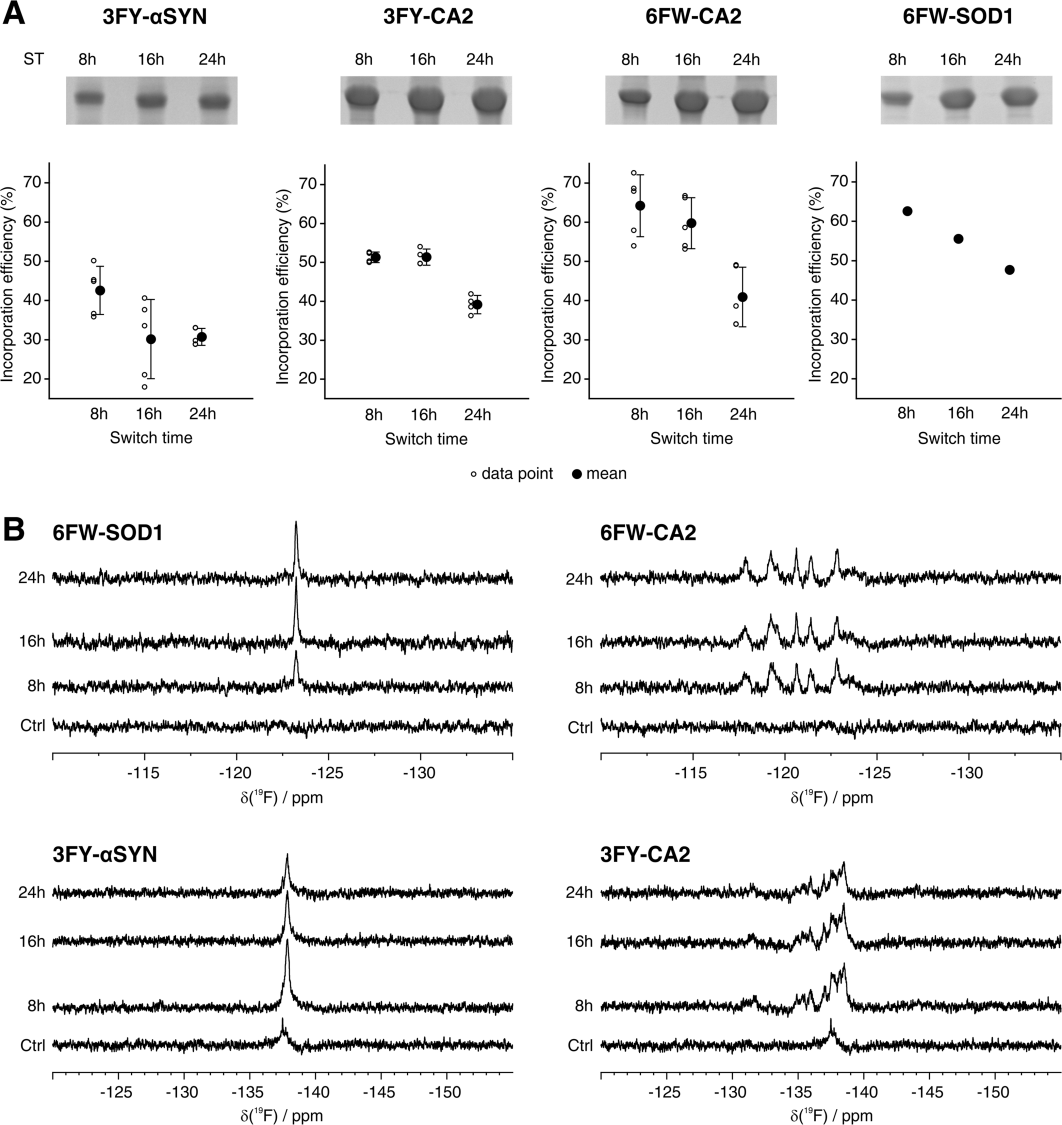
Effect of different medium switch times on incorporation efficiency, protein expression levels, and ^19^F NMR signal intensities. (A) SDS-PAGE analysis of soluble cell lysates obtained from small-scale expression (top) of 3FY-αSYN, 3FY-CA2, 6FW-CA2, and 6FW-SOD1, and plots of LC-MS data of the corresponding gel bands (bottom). Each data point in the plots presents the percentage of fluorine incorporation in a peptide containing a single target residue (see also Table S2). The means and standard deviations of each set of data points are also shown. Fluorine incorporation in 6FW-SOD1 is estimated from a single peptide. ST: switch time. (B) 1D ^19^F NMR spectra of cells expressing 6FW-SOD1 (top left), 6FW-CA2 (top right), 3FY-αSYN (bottom left), and 3FY-CA2 (bottom right) at different ST. Ctrl: control spectrum acquired on HEK293T cells transfected with pHL-empty.

The effects of different STs on protein expression levels, NMR signal intensities, and incorporation efficiency were evaluated by SDS-PAGE, in-cell ^19^F NMR, and MS, respectively (see the Materials and Methods section). By decreasing the ST, the incorporation efficiency for any tested conditions improved, as expected, however, the total protein levels were reduced (Figure 2A). As a result, 6FW incorporation efficiency after an 8 h ST was the highest for both SOD1 and CA2 (>60% from MS), but the protein yields were the lowest among the three selected ST conditions (Figure 2A and Table S2). In contrast, a 24 h ST gave rise to the highest yields, even though the incorporation efficiency was <50% for both proteins. The in-cell NMR signal intensities of 6FW-SOD1 and -CA2 in the tested conditions were overall similar (with the exception of 6FW-SOD1 at 8 h ST), indicating that higher protein yields compensated for the lower incorporation efficiency and vice versa (Figure 2B).

Unlike 6FW, the in-cell ^19^F NMR signal intensities of 3FY-CA2 and -αSYN, in samples obtained with different STs, were directly proportional to the incorporation efficiency (Figure 2). In the case of αSYN, the expression levels were not affected by shorter STs; thus, with an equivalent protein amount in cells, an 8 h ST offered the highest incorporation efficiency (∼43%) and therefore the highest signal intensity. On the other hand, 3FY-CA2 expression levels decreased slightly at shorter STs, yet the 8 h ST still provided the highest in-cell signal intensity. Intriguingly, the incorporation levels obtained from 8 h and 16 h STs did not differ from each other (∼51%, Figure 2A), possibly indicating an effect of 3FY incorporation on the expression rate of the protein.

The enzymatic digestions of 6FW-CA2, 3FY-CA2, and 3FY-αSYN generated peptides comprising two W or Y, respectively, that were informative about the completeness of endogenous incorporation (Figure S5 and Table S3). Overall, the percentages of both the mono-^19^F-peptide and the di-^19^F-peptide declined as the ST shortened. However, unlike 6FW incorporation, 3FY incorporation into CA2 and αSYN favored the mono-^19^F-peptide over the di-^19^F-peptide. This clearly indicates that the average number of fluorine atoms in a single protein molecule can vary greatly with the type of FAA used. Notably, despite the incomplete incorporation, MS analysis revealed the presence of FAAs at all expected positions of the target proteins, and therefore the ^19^F NMR spectra contain signals arising from each position. This was confirmed by the cell lysate spectrum of 3FY-αSYN, which matched the spectrum from the same protein produced in bacteria^43^ (Figure S4).

### Crystal Structure of 6FW-CA2

**Figure 3:**
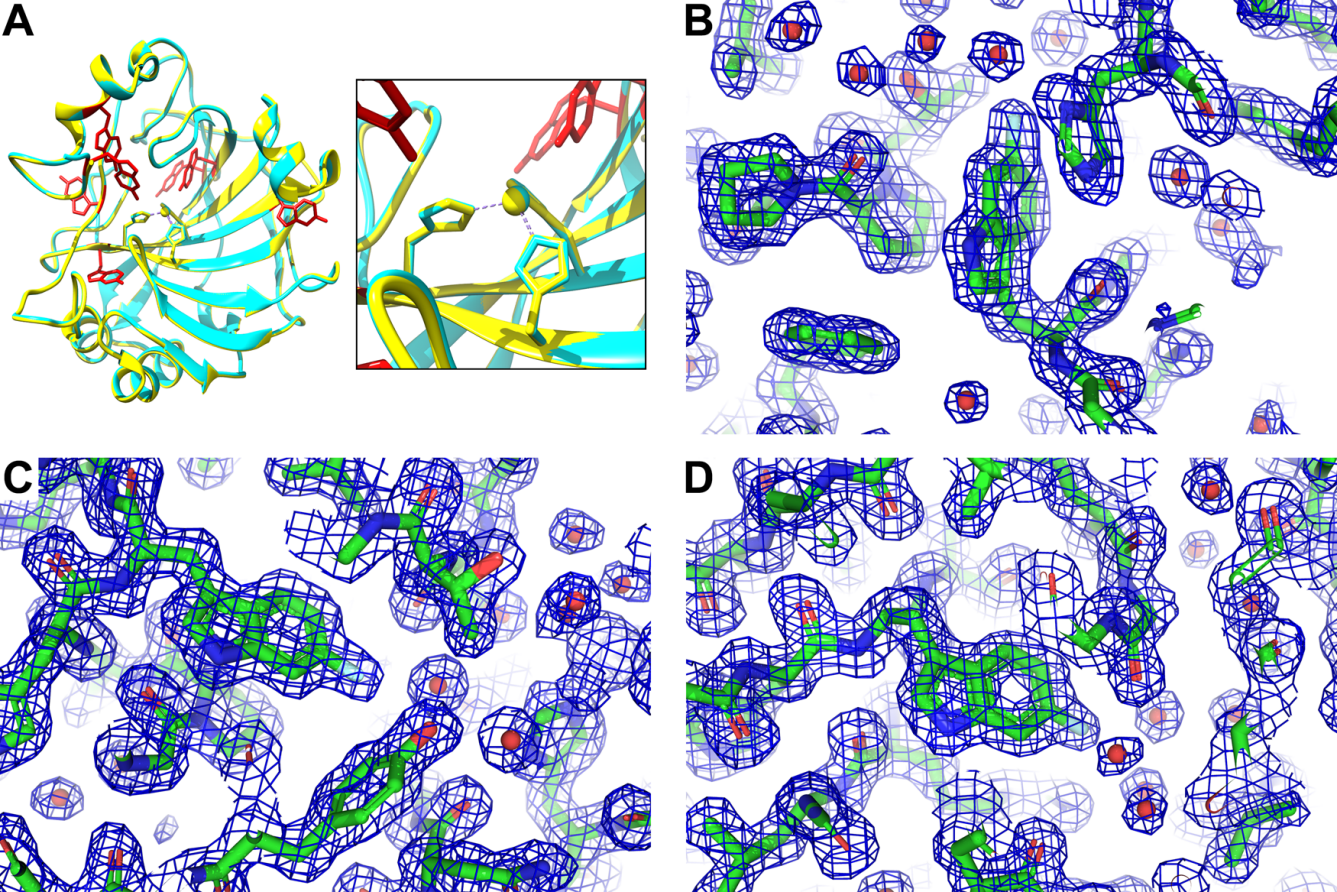
Structure of 6FW-CA2. (A) X-ray structure of 6FW-CA2 (PDB 8B29, cyan) superimposed to that of native, nonfluorinated CA2 (PDB 1CA2, yellow), showing negligible differences between the two structures. The inset shows the active site of the protein. Zn^2+^ is shown as sphere; Zn^2+^-coordinating histidines (cyan/yellow) and 6FW residues (red) are shown as sticks. (B–D) 2Fo-Fc maps of 6FW-CA2 contoured at 1σ (blue) showing the presence of fluorine atoms on Trp5 (B), Trp16 (C), and Trp123 (D). The polypeptide is shown as sticks, and water molecules are shown as spheres.

To evaluate the impact of FAA incorporation on protein structure, which could negatively affect the significance of ^19^F NMR data for drug screening applications, we determined the structure of 6FW-CA2 by X-ray crystallography. The protein was purified from human cells after a 24 h ST expression protocol and subsequently crystallized. The crystallographic structure (PDB 8B29) was obtained by molecular replacement at 1.7 Å resolution (Figure 3 and Table S4). The 3D structure of 6FW-CA2 is perfectly superimposable with those already deposited in the PDB and shares the same space group and cell parameters (backbone RMSD 0.21 Å). The presence of fluorine atoms in all seven tryptophan residues is evident in the electron density (Figure 3). The occupancy values of the fluorine atoms computed during refinement ranged from 0.4 to 0.6, in good agreement with the percentage of 6FW incorporation obtained by MS. Therefore, the incorporation of 6FW at up to seven positions in the CA2 polypeptide does not significantly affect any structural features of the protein, making 6FW-CA2 amenable for protein–ligand interaction studies by ^19^F in-cell NMR.

### Ligand Binding to CA2 by ^19^F In-Cell NMR

**Figure 4:**
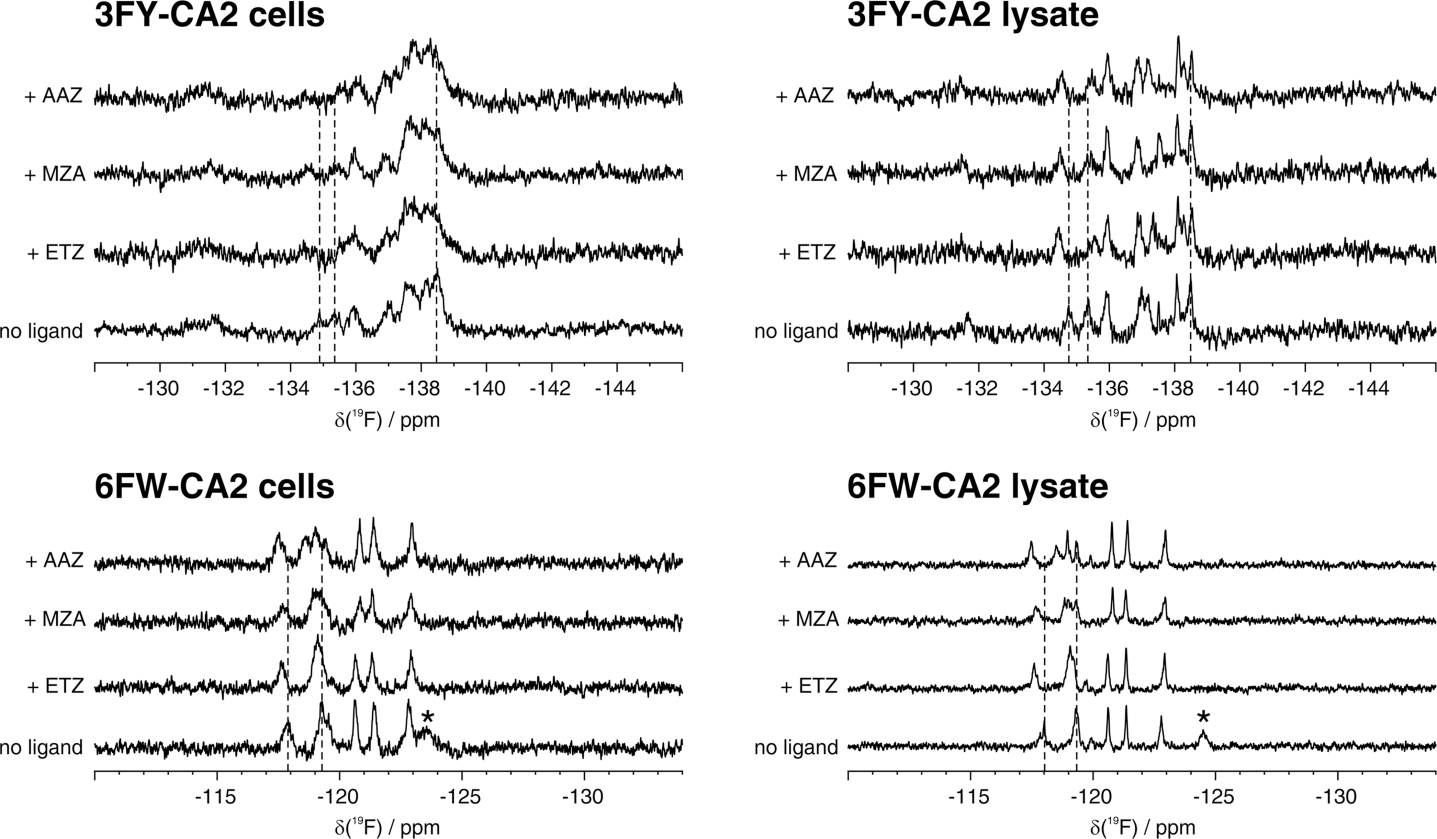
^19^F NMR of CA2 interacting with ligands in cells and soluble cell lysates. 1D ^19^F NMR spectra of cells (left) expressing 3FY-CA2 (ST = 8 h, top) and 6FW-CA2 (ST = 16 h, bottom) treated with different ligands, and of the corresponding lysates (right). No ligand: control spectra acquired on cells expressing CA2 with no ligand treatment, and corresponding lysates. ST: switch time. Peaks shifting upon binding are marked with dashed lines. *: disappearing signal upon ligand interaction.

The effects of protein–ligand interactions were then investigated on cells containing 3FY-CA2 and 6FW-CA2 expressed with the optimal STs, i.e., with 8 h ST and 16 h ST, respectively. At the end of expression, the cells were treated with 10 μM ethoxzolamide (ETZ), 10 μM methazolamide (MZA) for 1 h, or 50 μM acetazolamide (AAZ) for 2 h. These conditions were previously shown to lead to the complete binding of CA2 in HEK293T cells.^10^ Overall, both 3FY- and 6FW-CA2 samples presented distinct signal patterns from that of the unbound protein in the ^19^F in-cell NMR spectra, which were also confirmed in the cell lysates (Figure 4).

Upon the interaction of 3FY-CA2 with ligands, the signals at −134.4 and −135.3 ppm shifted by ca. 0.2–0.3 ppm, while the signal at −138.5 ppm decreased in intensity. By inspecting the lysate spectrum, the signals between −137.5 and −138.5 ppm showed splitting, suggesting that one of two overlapped peaks shifted due to ligand interaction. Compared to 3FY-CA2, 6FW-CA2 resulted in better resolved in-cell signals, hence facilitating the analysis of chemical shift changes. Ligand binding caused the disappearance of the CA2 signal at −123.5 and −124.5 ppm in the in-cell and lysate spectra, respectively, together with the shifting of the signal at −117.9 to −117.6 ppm. Remarkably, 6FW incorporation allowed distinguishing CA2 bound to different ligands, as the signal at −119.3 ppm either shifted to −119.0 ppm, broadened, or split into three separated peaks when 6FW-CA2 interacted with ETZ, MZA, or AAZ, respectively.

### Interaction between SOD1 and CCS-D2 by ^19^F In-Cell NMR

**Figure 5:**
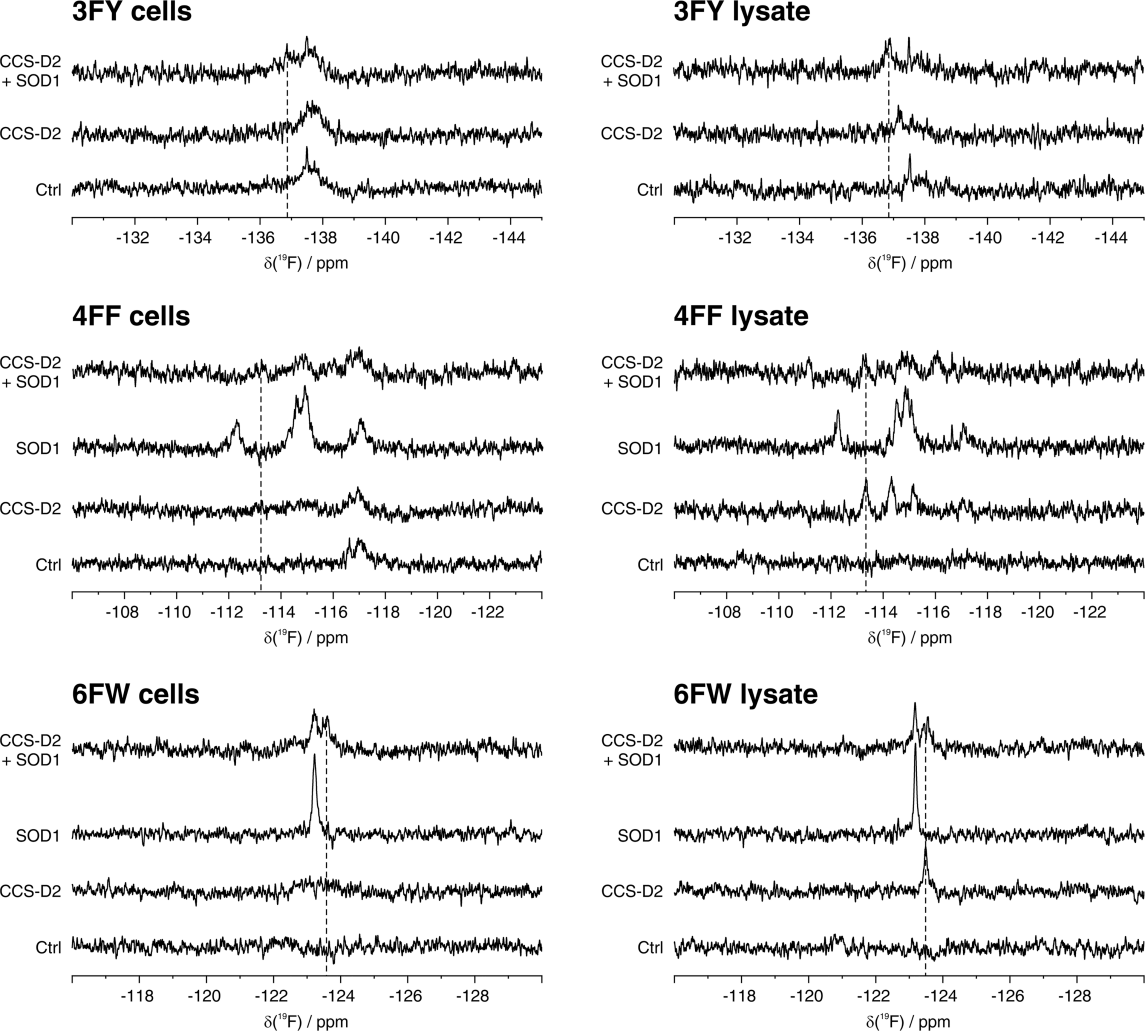
Interaction of CCS-D2 and SOD1 observed by ^19^F in-cell NMR. 1D ^19^F NMR spectra of cells (left) expressing 3FY- (top), 4FF- (middle), and 6FW- (bottom) SOD1 and CCS-D2 either alone or together (ST = 24 h), and of the corresponding lysates (right). Signals attributed to CCS-D2 in the complex with SOD1 are marked with dashed lines. Ctrl: control spectra acquired on cells transfected with pHL-empty and corresponding lysates.

The interaction between SOD1 and its structural homologue CCS-D2 was then investigated by ^19^F in-cell NMR. SOD1 and CCS-D2, which are both homodimers when expressed alone, are known to form a stable heterodimer when co-expressed in the human cell cytoplasm.^30^ Cells co-expressing both proteins incorporating 3FY, 4FF and 6FW, were analyzed together with the corresponding lysates. The resulting spectra showed clearly different patterns of signals compared to those of SOD1 and CCS-D2 alone, indicating an interaction between the two proteins (Figure 5). In addition, the overall line broadening was similar for both subunits of the heterodimer, whereas the signals of SOD1 were broader than those of the SOD1 homodimer, and those of CCS-D2 were sharper than those of the CCS-D2 homodimer. This marked change in linewidth indicates that the heterodimer still interacts with the cellular environment (likely through the CCS-D2 subunit), but to a lesser extent with respect to CCS-D2 alone. The presence of a stable heterodimer was also confirmed by ^19^F NMR spectra on the corresponding cell lysates, in which the signals of both subunits were better resolved (Figure 5). In the lysates, the linewidths of the heterodimer were comparable to those of SOD1 and CCS-D2 homodimers, consistent with the fact that cell lysis disrupted the interactions with the cellular environment (Figure 5).

## Discussion

The approach described above allowed the incorporation of fluorinated aromatic amino acids, 3FY, 4FF, 5FW, and 6FW, into various proteins (14–30 kDa) in human cells, and the direct observation by ^19^F in-cell NMR. Cells transiently expressing the protein of interest were supplemented with the desired FAA at an optimal post-transfection time point, and they efficiently utilized it for protein synthesis. Owing to the sensitivity of ^19^F NMR and the rarity of ^19^F in biological systems, the in-cell signals of interest were easily detected in 1D ^19^F NMR spectra with significantly low or absent cellular background. Notably, the approach is applicable to proteins known for having restricted tumbling motions in the cellular environment, such as CCS-D2^30^ and Nt-HSP90 [unpublished work], which would not be detectable in heteronuclear ^1^H-^15^N NMR spectra typically employed for in-cell NMR analysis. As the cellular background and line broadening often challenge the detection of target signals in in-cell studies, these observations demonstrated the great advantages of ^19^F NMR, suggesting significant potential of this approach.

Depending on the choice of FAA and on the expression conditions, the incorporation efficiency ranged between ∼30 and ∼60%. Such incomplete incorporation mainly arose from the fact that protein expression started a few hours after transfection, when the cells were still kept in nonfluorinated medium. As expected, shorter STs led to higher incorporation, however at the expense of the total expression level. An optimal ST is therefore required to maximize the amount of fluorinated protein, approximately given by total protein × incorporation efficiency. Notably, different optimal STs were found for 3FY and 6FW, suggesting that each FAA differently affects the protein expression rate over time. It is also worth noting that even though this method leads to a mixture of fluorinated and nonfluorinated proteins, only the fluorinated one is detected in the ^19^F NMR spectra; therefore, the fraction of protein expressed before the medium switch does not contribute to the final NMR spectrum.

We then showed that ^19^F in-cell NMR reported on the local chemical environment, hence providing relevant structural information. In general, we observed that the shape and dispersion of the ^19^F NMR signals were strongly dependent on the choice of FAA. This dependence was expected, due to the fact that different fluorinated analogs of a given amino acid introduce the fluorine atom at different positions in the protein structure, causing the fluorine to be exposed to different chemical environments. This is exemplified by the in-cell NMR spectra of incorporated tryptophan residues of CA2, showing distinctive signal patterns between 6FW and 5FW incorporations.

Additional effects on the shape and position of the ^19^F signals might also arise from the presence of side-chain rotamers that might fall into different NMR exchange regimes, depending on the local dynamics and would have a different impact on each FAA. For example, the 3FY ^19^F NMR signals would be likely affected by the existence of two different rotamers in folded proteins, related by a 180° rotation, leading to signal splitting and/or broadening. On the contrary, the fluorine atom in 4FF would not be affected by the ring flip, as it lies on the axis of rotation.

Finally, this approach can reveal conformational changes occurring upon interactions with ligands, as shown with the binding of ligands to fluorinated CA2, and with partner proteins, as observed with the heterodimer formation between SOD1 and CCS-D2. For this kind of applications, the choice of FAA labeling will depend on the position of the fluorine atom(s) relative to the site of interaction with the ligand or partner protein. ^19^F nuclei closer to the interaction site will likely experience larger chemical shift perturbations and therefore will be more sensitive reporters. In the case of CA2, the high number of tryptophan residues allowed probing several positions on the protein structure, some of which were clearly perturbed upon binding. However, in general, that might not be the case, as in other proteins, tryptophan residues may be less common and/or unaffected by the interaction. In such cases, possible solutions would include switching to a different FAA incorporation or introducing a point mutation at the desired position on the protein structure.

## Conclusions

In this work, we performed the endogenous incorporation of fluorinated amino acids into target proteins in human cells, which were then investigated directly in living cells by in-cell ^19^F NMR spectroscopy. To our knowledge, this is the first in-cell ^19^F NMR application where proteins are expressed and fluorinated directly in human cells. As such, it offers a robust and cost-effective alternative to previous approaches relying on the delivery of exogenous proteins, where a large amount of recombinant fluorinated protein would be needed. The methodology was demonstrated on several cytoplasmic proteins and can be applied to incorporate different fluorinated analogs of aromatic amino acids. The target proteins were successfully detected in the ^19^F in-cell NMR spectra, even with suboptimal hardware, with minor or negligible interference from ^19^F cellular background. The methodology was applied to detect the binding of small organic molecules to an intracellular target, CA2, and to observe the interaction between a metalloenzyme, SOD1, and the recognition domain of the metallochaperone CCS, responsible for its maturation. Notably, ^19^F NMR allowed the detection of very broad signals arising from slow-tumbling intracellular proteins, CCS-D2 and Nt-HSP90, which could not be detected by ^1^H-^15^N NMR experiments. The slow-tumbling behavior of many intracellular proteins hampers the widespread application of solution NMR to in-cell studies, and we believe that ^19^F has the potential to overcome such limitation, also thanks to the development of novel pulse schemes suitable for large complex molecules.^44,45^ We predict that ^19^F NMR detection, regardless of the choice of sample preparation approach, will be instrumental in the structural and functional studies of challenging protein targets in human cells.

## Supporting Information Available

SDS-PAGEs of protein expression tests as a function of FAAs dose and time; ^19^F NMR spectra of extracellular media; spectral assignment of 3FY-αSYN ^19^F signals; incorporation efficiency in peptides with two FAAs (Figures S1–S5) and expression levels of fluorinated proteins; percentages of fluorine incorporation in each peptide measured by mass spectrometry; and X-ray data collection and refinement statistics for PDB entry 8B29 (Tables S1–S4) (PDF)
