# Supplementary material for: Direct Expression of Fluorinated Proteins in Human Cells for 19F In-Cell NMR Spectroscopy
Source: J Am Chem Soc. 2023 Jan 5;145(2):1389–99. doi: 10.1021/jacs.2c12086 (PMC9853860; doi:10.1021/jacs.2c12086)
Supplement: Supplementary file 1 — ja2c12086_si_001.pdf [file ja2c12086_si_001.pdf]

Supporting Information

Direct expression of fluorinated proteins in human cells for ^{19}F in-cell NMR spectroscopy

Lan B. T. Pham[1], Azzurra Costantino[1], Letizia Barbieri[1,2], Vito Calderone[1,3], Enrico Luchinat[2,4]*, Lucia Banci[1,2,3]*

[1] CERM – Magnetic Resonance Center, Università degli Studi di Firenze, Via Luigi Sacconi 6, 50019 Sesto Fiorentino, Italy;
[2] Consorzio Interuniversitario Risonanze Magnetiche di Metallo Proteine – CIRMMP, Via Luigi Sacconi 6, 50019 Sesto Fiorentino, Italy;
[3] Dipartimento di Chimica, Università degli Studi di Firenze, Via della Lastruccia 3, 50019 Sesto Fiorentino, Italy;
[4] Dipartimento di Scienze e Tecnologie Agro-Alimentari, Alma Mater Studiorum – Università di Bologna, Piazza Goidanich 60, 47521 Cesena, Italy;
* Corresponding Authors: Enrico Luchinat: enrico.luchinat@unibo.it; Lucia Banci: banci@cerm.unifi.it.

Supplementary Figures S1-S5

Supplementary Tables S1-S4

Supplementary Figures

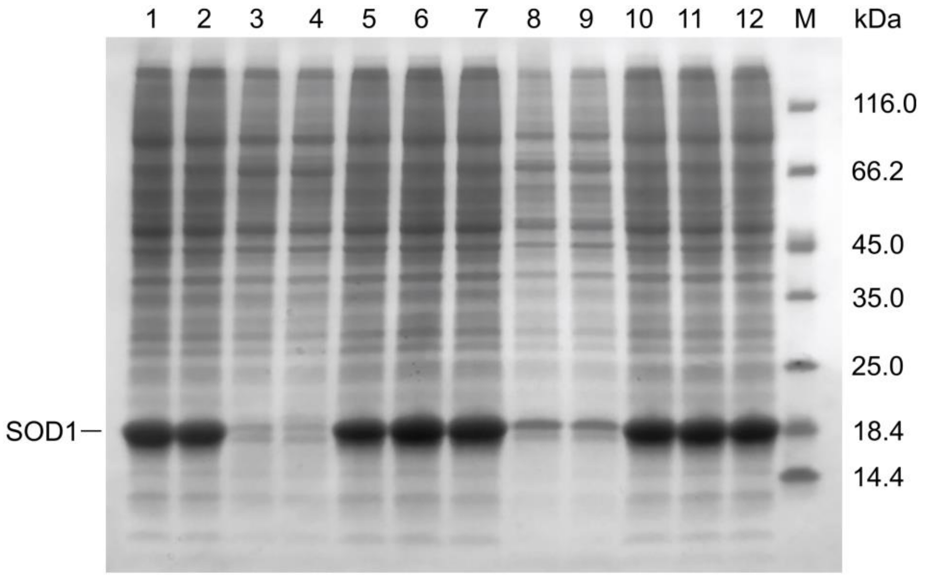

Figure S1. Expression of SOD1 in HEK293T cells supplemented with FAA-containing media at different doses and times. Coomassie-stained SDS-PAGE of lysates from HEK293T cells expressing SOD1 in different media formulations with different FAA medium switch times (ST): 1) commercial DMEM; 2) in-house DMEM without FAAs; 3) in-house DMEM supplemented with 1x 4FF at ST = 0 h; 4) in-house DMEM supplemented with 2x 4FF at ST = 0 h; 5) in-house DMEM supplemented with 0.5x Phe + 0.5x 4FF at ST = 0 h; 6) in-house DMEM supplemented with 2/3x Phe + 1/3x 4FF at ST = 0 h; 7) in-house DMEM supplemented with 1x 4FF at ST = 24 h; 8) in-house DMEM supplemented with 1x 6FW at ST = 0 h; 9) in-house DMEM supplemented with 2x 6FW at ST = 0 h; 10) in-house DMEM supplemented with 0.5x Trp + 0.5x 6FW at ST = 0 h; 11) in-house DMEM supplemented with 2/3x Trp + 1/3x 6FW at ST = 0 h; 12) in-house DMEM supplemented with 1x 6FW at ST = 24 h; M = marker. 1x, 2x, etc. indicate the dose of FAA with respect to the commercial DMEM formulation.

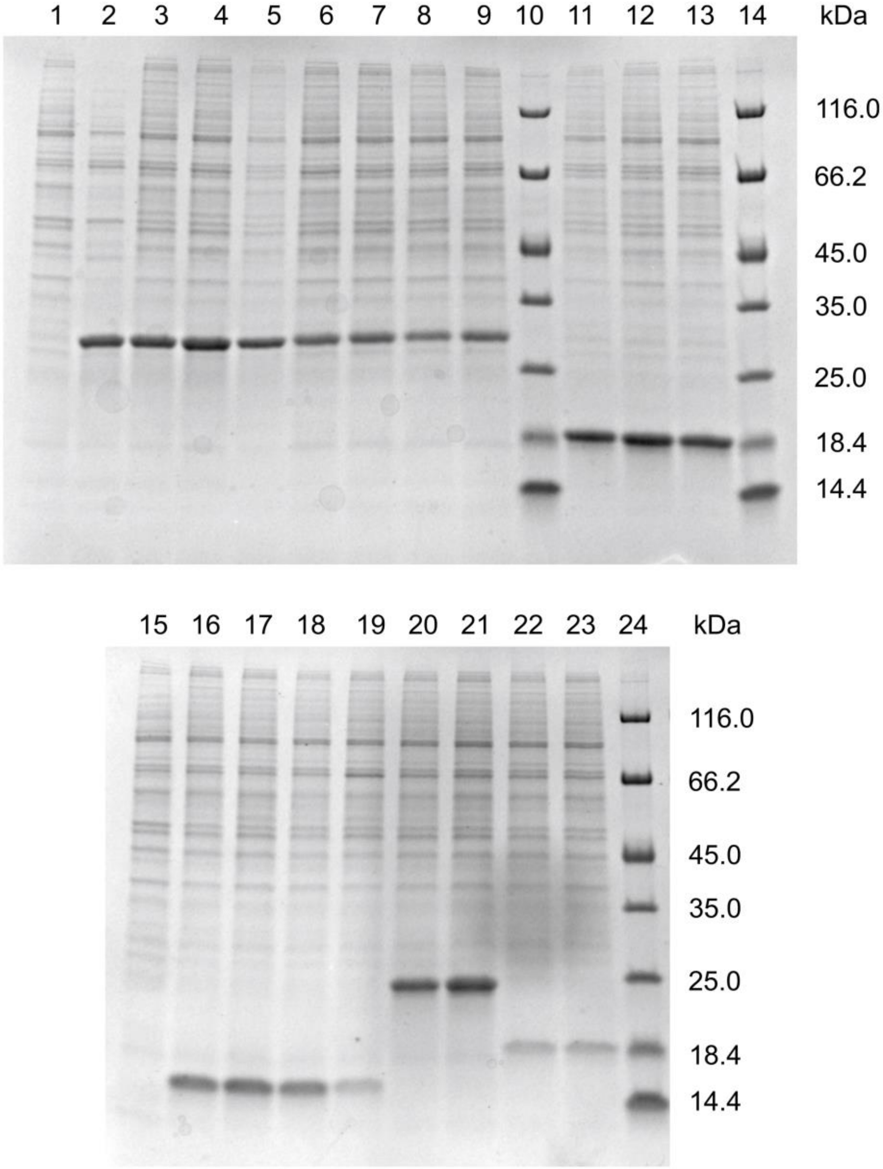

Figure S2. Expression of different proteins in HEK293T cells supplemented with different FAAs. Coomassie-stained SDS-PAGE of lysates (diluted 1:20) from HEK293T cells expressing different proteins in in-house DMEM supplemented with different FAAs at ST = 24 h: 1) pHL-empty; 2) CA2 with 6FW; 3) CA2 with 5FW; 4) CA2 with 4FF; 5) CA2 with 3FY; 6) Nt-HSP90 with 6FW; 7) Nt-HSP90 with 5FW; 8) Nt-HSP90 with 4FF; 9) Nt-HSP90 with 3FY; 10) MW marker; 11) SOD1 with 6FW; 12) SOD1 with 5FW; 13) SOD1 with 4FF; 14) MW marker; 15) pHL-empty; 16) CCS-D2 with 6FW; 17) CCS-D2 with 5FW; 18) CCS-D2 with 4FF; 19) CCS-D2 with 3FY; 20) DJ-1 with 4FF; 21) DJ-1 with 3FY; 22) αSYN with 4FF; 23) αSYN with 3FY; 24) MW marker.

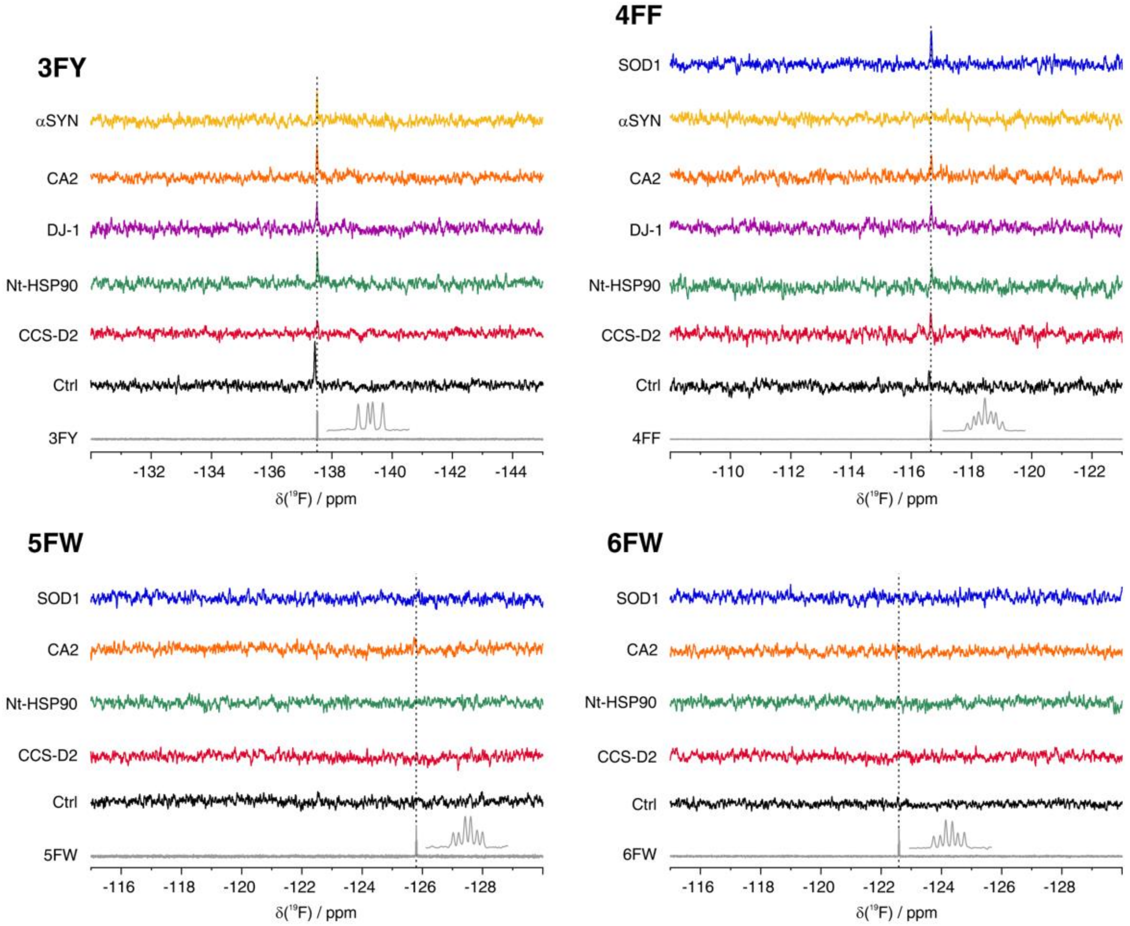

Figure S3. ^{19}F NMR spectra of the extracellular medium after the in-cell NMR experiments. 1D ^{19}F NMR spectra of the extracellular medium recorded after each in-cell NMR experiment shown in Figure 1. 3FY (top left), 4FF (top right), 5FW (bottom left) and 6FW (bottom right). The spectra show that no leakage of fluorinated proteins occurred during the ^{19}F in-cell NMR analysis. The spectra of pure FAAs in PBS (grey) are shown as reference; each inset shows the multiplet structure arising from ^{19}F-^{1}H J-couplings. The chemical shift of each FAA is marked with a dotted line.

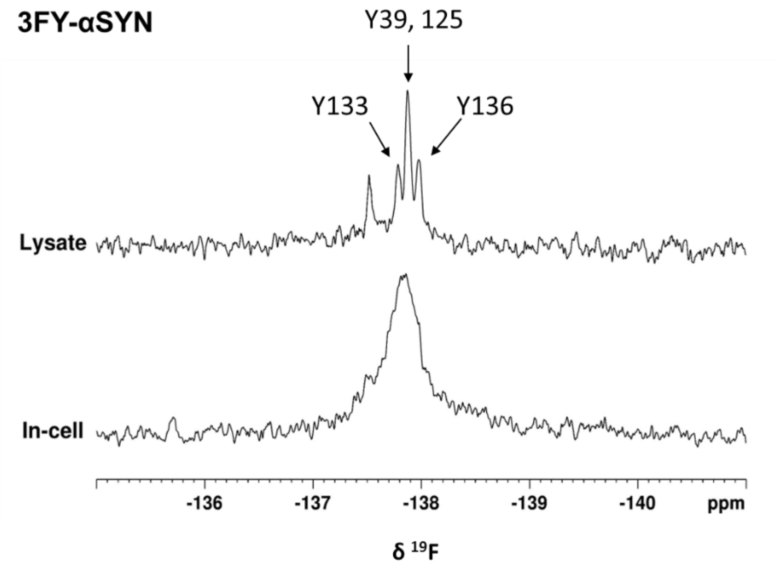

Figure S4. ^{19}F NMR spectra of 3FY-αSYN. In-cell (bottom) and cell lysate (top) NMR spectra of 3FY-αSYN expressed with a ST of 8h. The peaks in the lysate spectrum are labeled according to a previously reported assignment.[1]

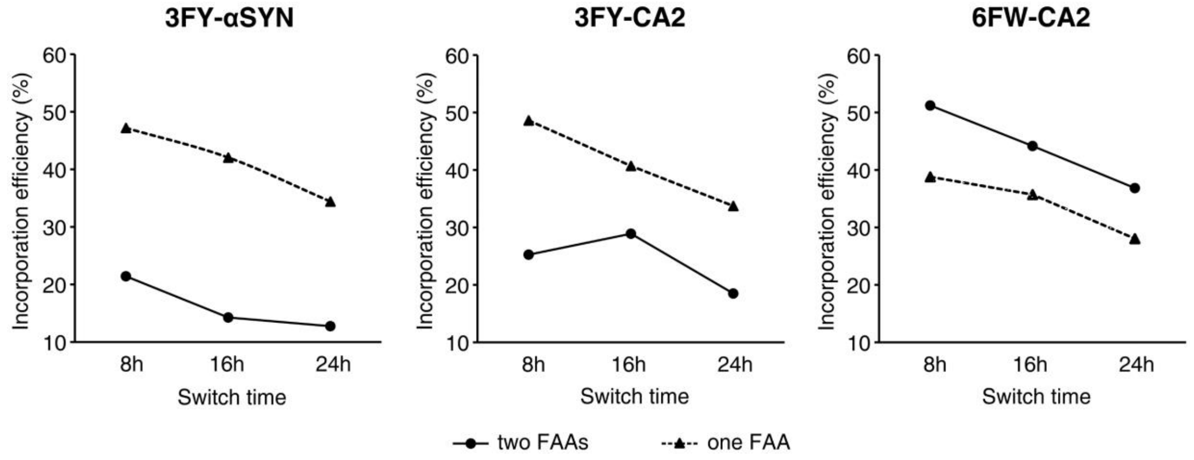

Figure S5. The incorporation efficiency assessed by peptides of 3FY-αSYN, -CA2 and 6FW-CA2, containing two target residues (W or Y) with one or two FAA. The incorporation efficiency is presented by the percentages of peptides with one or two incorporation(s) (Table S3). For peptide 126-140 of 3FY-αSYN, the average percentages of peptide with and without post-translational modification were achieved and plotted. FAA: fluorinated amino acid.

Supplementary Tables

Table S1. Expression levels of fluorinated proteins in HEK293T cells. Concentrations of fluorinated proteins in 150 µL lysates obtained from one T75 flask of HEK293T cells, estimated by densitometry analysis of Coomassie-stained SDS-PAGE using BSA and CA2 as protein standards. A ~10% sample-to-sample variability is estimated due to differences in number of cells and transfection efficiency.

Proteins	Protein concentration (µM)			
	**3FY**	**4FF**	**6FW**	**5FW**
**αSYN**	120	100	-	-
**CA2**	200	230	200	200
**CCS-D2**	230	270	260	210
**DJ-1**	260	220	-	-
**Nt-HSP90**	110	110	110	110
**SOD1**	-	360	300	340

Table S2. The percentages of FAA incorporation in peptides containing one target residue (W or Y) at different switch times analyzed by mass spectrometry. The target residues in each peptide sequence are underlined and post-translational modifications are labeled in red. FAA: fluorinated amino acid.

Proteins	Enzymatic digestion	Peptide sequences	Positions	% of peptide containing FAA		
				8 h	16 h	24 h
**6FW-SOD1**	Trypsin	VWGSIK	32-37	62.62	55.57	47.68
**6FW-CA2**	Trypsin	SHHWGYGK	2-9	58.32	57.30	35.71
		HNGPEHWHK	10-18	67.08	53.36	40.47
		LIQFHFHWGSLDGQGSEHTVDK	90-111	51.77	54.09	40.93
		YAAELHLVHWNTK	114-126	68.63	66.24	48.9
		LNFNGEGEPEELMVDNWRPAQPLK	228-251	67.14	62.38	47.38
**3FY-CA2**	Trypsin	GGPLDGTYR	81-89	50.29	49.67	38.56
		YAAELHLVHWNTK	114-126	52.33	51.99	39.95
		KYAAELHLVHWNTK *(one missed cleavage)*	113-126	52.63	54.00	41.88
		YGDFGK	127-137	49.99	49.70	36.27
**3FY-aSYN**	Chymotrypsin	SKAKEGVVAAAEKTKQGVAEAAGKTKEGVLY *(one missed cleavage)*	9-39	36.86	23.52	29.48
		VKKDQLGKNEEGAPQEGILEDMPVDPDNEAY *(two missed cleavages)*	95-125	43.50	36.82	34.15
		VKKDQLGKNEEGAPQEGILEDMPVDPDNEAY *(two missed cleavages with oxidized methionine)*	95-125	50.35	40.04	32.43
		EDMPVDPDNEAY	114-125	36.68	17.95	28.87
		EDMPVDPDNEAY *(with oxidized methionine)*	114-125	45.29	33.56	28.85

Table S3. The percentages of fluorine incorporation in peptides containing two target residues (W or Y) at different switch times analyzed by mass spectrometry. The percentages were calculated using the sum of no-FAA, one-FAA and two-FAA containing molecular ions. FAA: fluorinated amino acid.

Proteins	Enzymatic digestion	Peptide sequences	Positions	Incorporation(s)	% peptide containing FAA		
					8h	16h	24h
**6FW-CA2**	Trypsin	GLLPESLDYWTYPGSLTTPPLLECVTWIVLKE PISVSSEQVLK *(one missed cleavage)*	182-224	1	38.28	36.21	27.59
				2	51.98	45.17	38.41
**3FY-CA2**	Trypsin	YDPSLKPLSVSYDQATSLR	40-58	1	65.04	57.28	41.39
				2	49.14	48.78	27.93
**3FY-aSYN**	Chymotrypsin	EMPSEEGYQDYEPEA (two missed cleavages)	126-140	1	59.98	49.32	40.02
				2	40.58	24.54	19.58
		EMPSEEGYQDYEPEA (two missed cleavages with oxidized methionine)	126-140	1	60.14	48.78	38.86
				2	40.60	24.72	19.36

Table S4. Data collection and refinement statistics of PDB 8B29. Statistics for the highest-resolution shell are shown in parentheses.

Parameter	Value
**Wavelength**	1.541
**Resolution range**	28.64 - 1.701 (1.761 - 1.701)
**Space group**	$P2_1$
**Unit cell**	42.2 41.22 71.96 90 104.33 90
**Unique reflections**	23397 (1385)
**Multiplicity**	5.7 (3.7)
**Completeness (%)**	91.8 (52.03)
**Mean I/sigma(I)**	10.68 (2.08)
**Wilson B-factor**	13.09
**R-merge**	0.1235 (0.7579)
**CC1/2**	0.995 (0.588)
**Reflections used in refinement**	23395 (1385)
**Reflections used for R-free**	1169 (69)
**R-work**	0.1624 (0.2399)
**R-free**	0.2096 (0.2877)
**Number of non-hydrogen atoms**	2354
Macromolecules	2065
Ligands	1
Solvent	288
**Protein residues**	258
**RMS(bonds)**	0.014
**RMS(angles)**	1.35
**Ramachandran outliers (%)**	0.00
**Rotamer outliers (%)**	0.00
**Clashscore**	2.22
**Average B-factor**	15.38
Macromolecules	14.35
Ligands	10.72
Solvent	22.85

References

(1) Li, C.; Lutz, E.A.; Slade, K.M.; et al. 19F NMR Studies of α-Synuclein Conformation and Fibrillation. *Biochemistry* **2009**, *48*, 8578-8584.
